# The influence of visual perspective on the somatosensory steady-state response during pain observation

**DOI:** 10.3389/fnhum.2013.00849

**Published:** 2013-12-09

**Authors:** Dora L. Canizales, Julien I. A. Voisin, Pierre-Emmanuel Michon, Marc-André Roy, Philip L. Jackson

**Affiliations:** ^1^École de Psychologie, Université LavalQuébec, QC, Canada; ^2^Centre de Recherche de l’Institut Universitaire en Santé Mentale de QuébecQuébec, QC, Canada; ^3^Centre Interdisciplinaire de Recherche en Réadaptation et Intégration SocialeQuébec, QC, Canada; ^4^Département de Réadaptation, Université LavalQuébec, QC, Canada

**Keywords:** pain observation, visual perspective, empathy, electroencephalography, somatosensory steady-state

## Abstract

The observation and evaluation of other’s pain activate part of the neuronal network involved in the actual experience of pain, including those regions subserving the sensori-discriminative dimension of pain. This was largely interpreted as evidence showing that part of the painful experience can be shared vicariously. Here, we investigated the effect of the visual perspective from which other people’s pain is seen on the cortical response to continuous 25 Hz non-painful somatosensory stimulation (somatosensory steady-state response: SSSR). Based on the shared representation framework, we expected first-person visual perspective (1PP) to yield more changes in cortical activity than third-person visual perspective (3PP) during pain observation. Twenty healthy adults were instructed to rate a series of pseudo-dynamic pictures depicting hands in either painful or non-painful scenarios, presented either in 1PP (0–45° angle) or 3PP (180° angle), while changes in brain activity was measured with a 128-electode EEG system. The ratings demonstrated that the same scenarios were rated on average as more painful when observed from the 1PP than from the 3PP. As expected from previous works, the SSSR response was decreased after stimulus onset over the left caudal part of the parieto-central cortex, contralateral to the stimulation side. Moreover, the difference between the SSSR was of greater amplitude when the painful situations were presented from the 1PP compared to the 3PP. Together, these results suggest that a visuospatial congruence between the viewer and the observed scenarios is associated with both a higher subjective evaluation of pain and an increased modulation in the somatosensory representation of observed pain. These findings are discussed with regards to the potential role of visual perspective in pain communication and empathy.

## Introduction

Seeing pain in other people is an experience susceptible to trigger responses akin those felt when we hurt ourselves. Indeed, several neuroimaging studies found a partial overlap between cerebral circuits involved during actual experience of pain (known as the pain matrix) and during the observation of other’s pain (for a recent review see Fan et al., 2011; Lamm et al., 2011). More specifically, the observation of other’s pain engages in a similar way some of the neuronal systems subserving the sensory (e.g., somatosensory cortices) and the affective components (e.g., insula, anterior cingulate cortex) of self pain perception (Fitzgibbon et al., 2010; Corradi-Dell’Acqua et al., 2011) . The activation of these regions suggests, indirectly, that observing pain produces a fine-grained multidimensional mental representation of the other’s pain even in the absence of somatosensory input. Even though this mental representation of pain enables an observer to partially share the subjective experience of other’s pain, the extent of this sharing mechanism can vary according to different factors (Coll et al., 2011). Note that the specificity of the nociceptive cerebral representation itself (signature; Wager et al., 2013) is currently being debated and the extent to which this representation also codes for the pain of others (Krishnan et al., 2013) and social pain (Iannetti et al., 2013) appears to be more limited than initially thought.

Nevertheless, recent neuroimaging studies have provided strong evidence that observation of pain can involve the somatosensory cortex, known to contribute to sensory processing of noxious stimuli (Bufalari et al., 2007; Lamm et al., 2007b; Cheng et al., 2008; Betti et al., 2009; Han et al., 2009; Yang et al., 2009; Voisin et al., 2011a). Moreover, the recruitment of sensory processes of pain is distinctly demonstrated by a decrease of the somatosensory response during pain observation (Cheng et al., 2008; Voisin et al., 2011a; Marcoux et al., 2013). Although the involvement of the sensory cerebral circuits during pain observation has been repetitively demonstrated (see Keysers et al., 2010; Lamm et al., 2011, for reviews), the variables that modulate these sensory processes remain unclear.

A growing body of evidence supports the idea that somatosensory activity is influenced by *perspective taking* (*PT*; Ruby and Decety, 2001, 2003, 2004; Jackson et al., 2006a,b) PT can be defined as the ability to adopt someone else’s point of view in order to understand their situation (Decety et al., 2006). This ability represents an essential component of empathy, which refers to the faculty to understand and to share other’s emotions and feelings and to respond appropriately (Decety et al., 2006). Studies generally distinguish cognitive PT, which requires the individual to imagine being the other person (e.g., Ruby and Decety, 2003; Jackson et al., 2006b; Dosch et al., 2010) from visual PT, which involves seeing a scene or a situation from different angles (e.g., Jackson et al., 2006a; Kessler and Thomson, 2010). Regarding cognitive PT, several studies demonstrated that thinking about oneself in a specific situation generates different behavioral and cerebral responses than imagining another person in the same situation (Ruby and Decety, 2004; Jackson et al., 2005, 2006b; Lamm et al., 2007a, 2008; Li and Han, 2010) While self-perspective requires fast and automatic processes, which are more related to agency (i.e., ability to attribute the origin of an action), adopting the perspective of others appears to engage more deliberate and regulatory mechanisms (van der Heiden et al., 2013).

Visual PT generally results from a mental rotation of one’s own perspective toward the other’s perspective in order to consider the spatial information from the other’s viewpoint that may be different from the subject’s one (Kozhevnikov and Hegarty, 2001; Kessler and Thomson, 2010). Visual PT provides crucial spatial information that enables a person to appropriately conduct social interaction and understand other’s mental states (Langdon and Coltheart, 2001; Kaiser et al., 2008; Lambrey et al., 2008; Kessler and Thomson, 2010). The manipulation of the visual perspective is broadly used in cinematography (e.g., subjective/objective camera), particularly in horror movies and video games (e.g., first/third-person games) in order to generate the feeling in the spectator of sharing the point of view of the character.

Behavioral studies generally report faster reaction time and increased accuracy performance when an object or an action is seen in a *first-person* visual perspective (1PP) (i.e., seeing a situation from the onlooker’s viewpoint) compared to a *third-person* visual perspective (3PP) (i.e., seeing a situation presented in someone else’s viewpoint) (Jackson et al., 2006a; Kaiser et al., 2008). Jackson et al. (2006a) demonstrated that seeing or imitating actions performed in the 1PP yielded stronger sensorimotor activation in comparison to the 3PP. This supports the assumption that adopting 1PP generates more robust sensorimotor representation of the action in the onlooker’s brain that may be close to actual execution of the action. 3PP also seems to involve specific neuronal processes associated with spatial transformations (Jackson et al., 2006a; Kaiser et al., 2008; Callan et al., 2012), visual motion perception (Bundo et al., 2000; de Lussanet et al., 2008), and executive functions such as inhibition and attention (Hampshire et al., 2010; Dodds et al., 2011). Altogether, these findings show that adopting 1PP and 3PP requires distinct neuronal processes: the former may be more associated with automatic embodiment (resonance) and the latter with cognitive functions such as visuospatial processing and inhibition.

The main objective of this study was to determine if the point of view of the observer (visual perspective) can specifically modulate the behavioral and cerebral responses to painful visual stimuli. To do so, we compared the modulation of the somatosensory steady-state response (SSSR) during the observation of painful visual stimuli depicted in a 1PP and 3PP. Firstly, we hypothesized that participants would attribute higher pain ratings to painful pictures depicted in the 1PP. Secondly, we suggested that seeing the pictures would produce an automatic decrease of the SSSR amplitude (i.e., *initial gating effect*), which will occur mainly over the left parietal cortex as this region was previously found to be more responsive to steady-state somatosensory stimulation (Voisin et al., 2011a,b; Marcoux et al., 2013). Thirdly, we predicted that this SSSR modulation would be *a priori* greater when painful situations were presented in a first-person compared to a 3PP (i.e., *visual perspective effect*). Finally, we have also examined the association between the SSSR response and self reported measures of different components of empathy.

## Methods

### Participants

The sample was composed of 20 healthy right-handed Caucasian volunteers (nine men, mean age = 25 ± 5 years). Participants had no history of neurological, psychiatric or pain related disorders, and visual acuity was normal or corrected. This participant had not completed the whole experiment and quit due to discomfort during the task. The study was approved by the Research Ethic Committee of the Institut de Réadaptation en Déficience Physique de Québec. Participants gave written informed consent and received a small monetary compensation for their participation.

## Material

### Visual stimuli and experimental procedure

Pseudo-dynamic visual stimuli presented the right hand of adult Caucasian (half male, half female) displayed in 12 different everyday life scenarios (e.g., cutting food with a knife). These scenarios were shown either in a first (1PP: arm at 0°–45° angle) or third person visual perspective (3PP: arm at ∼180° angle). The scenarios ended in a painful or nonpainful situation (Pain vs. NoPain condition). There were 12 different scenarios displaying two types of visual perspective (1PP and 3PP), two pain levels (Pain and Nopain), and two models’ sex (Male and Female), giving a total of 96 different visual stimuli. Visual stimuli were perceived as dynamic because they were composed of a sequence of three pictures, respectively displayed for 750, 250 and 1500 ms for a total length of 2500 ms. The participants could see the type of visual perspective from the first picture on, but the painful vs. nonpainful outcome appeared only in the third picture. This was done to equate pain anticipation across conditions. Note that the motor and sensory components in the stimuli varied (hand moving away from a situation, danger approaching hand) but these were distributed across conditions so as to avoid bias. This relative heterogeneity improves ecological validity and reduces repetitiveness, which could lead to habituation effects.

**Figure 1:**
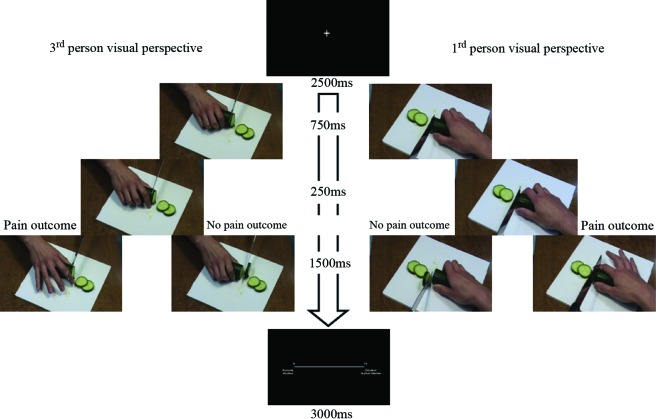
**Illustration of the task design: sequences of three pictures showing painful or nonpainful scenarios were displayed in a first (1PP arm at 0°–45° angle; right-hand side of the figure) or third person visual perspective (3PP: arm at ~180° angle; left-hand side of the figure).** Visual stimuli were presented in sequence with variable timing (see text) in order to induce a pseudo-dynamic effect. The type of visual perspective was perceptible from the first picture on, and the painful or nonpainful outcome appeared only in the third picture. The pain pictures were followed by a rating scale to remind participants to provide their response aurally.

The experimental task, scripted in E-Prime (Version 2.0, Psychology Software Tools, Inc.), contained eight blocks of 24 trials in which the four conditions (two Pain levels [pain, no pain] × two Perspectives [first, third]) were presented six times each in random order. Each trial comprised a fixation cross (2500 ms), the dynamic visual stimulus (2500 ms) and a verbal numerical rating scale ranging from 0 (No Pain) to 10 (Worst Pain) (3000 ms) (see Figure 1). The total length of a block was 4 min. The gender of the person on the visual stimuli was equally and randomly distributed in each block. The participants were instructed to verbally rate the intensity of pain observed after each picture once the numeric scale appeared on the screen. To make sure that the instructions were well understood, the participants also completed a short practice session (12 trials) before the experimental session. A trial sample is shown in Figure 1.

Throughout each trial block, a continuous and nonpainful mechanical stimulation (25 Hz) provided by a custom-made cylinder-shaped vibrotactile stimulator (10 cm long, 3 cm diameter) held in the participants’ right hand. The right hand rested on an armrest and electromyographic activity was recorded (MP150 system, Biopac Inc.) with Ag-AgCl surface electrodes positioned in bipolar configuration over the first dorsal interosseus muscle (FDI). Participants were told to not contract the stimulator with their hand during the experimental session. EMG activity was visually examined to monitor that participants did not change the grip significantly on the stimulator.

### Interpersonal Reactivity Index questionnaire

A French translation of the Interpersonal Reactivity Index (IRI; Davis, 1980) self-report questionnaire was administrated to the participants. The IRI is a measure of dispositional empathy in which participants had to determine the level of agreement or disagreement about thoughts and feelings in a variety of situations using a 5-point Likert-type scale. The IRI contains four 7-item subscales: the Empathic Concern (EC) scale measures the tendency to experience feelings of sympathy and compassion for others; the Personal Distress (PD) scale evaluates the inclination to feel discomfort and helplessness in response to other’s people distress; the PT scale assesses the propensity to adopt other person’s point of view; and the Fantasy (F) scale measures the tendency to imagine oneself into fictional situations. The score on each subscale is used as independent measure of four abilities related to empathy.

### EEG

During the EEG data acquisition, the participants were comfortably seated on a chair with armrest in a quiet dark room. An EEG helmet with 124 + 4 Ag/AgCl electrodes contacting scalp surface by way of saline-soaked sponges (Electrical Geodesic Inc., OR, USA) was used to record the cerebral activity. The sampling rate was set at 500 Hz and the electrodes impedances were kept below 50 kΩ.

## Analyses

### Behavioral analyses

First, trials where participants gave an incorrect response were discarded from subsequent analyses. An incorrect response occurred when a participant rated a painful picture as nonpainful (rating of zero) and a nonpainful picture as painful (ratings of 1 to 10). This procedure was applied to make sure that the participants have correctly categorized the visual stimuli and were paying attention to the task. The analyses were conducted on correct painful trials only. A repeated measure ANOVA (Pain, Perspective and Block conditions) was computed to confirm that evaluation of painful visual stimuli were not influenced by habituation across blocks. Ratings for the nonpainful pictures were not kept for the subsequent analyses. A paired *t*-test was calculated for the difference between mean rating of painful pictures presented in the 1PP and 3PP. All statistical analyses were computed with the SPSS v.13 software (SPSS Inc., Chicago, IL, USA).

### EEG pre-processing and analyses

The EEG analyses were ran using the locally-developed software ELAB plus the ELAN-Pack (Aguera et al., 2011) and MATLAB software (version 6.5; The Math-Works inc., Natick, MA). Note that trials that were removed from the behavioral data (see above) were also rejected from the EEG data. Visual inspection of very high level of noisy data led to the rejection of all data from a second participant who, furthermore, demonstrated important signs of anxiety and agitation relatively to the EEG apparatus. The EEG signal was cleaned from blinks, muscle activity, fast baseline shift, and high inter-electrodes impedance. More specifically, any 100 ms-long sample was rejected if it included one of these events: (i) in the same electrode channel, the scalp potential exhibited variation over 50 μV within a 10 ms time window; (ii) in the same electrode channel, the energy content was more than 500 μV^2^ in the 60–100 Hz band; or (iii) 800 μV^2^ in the 23–27 Hz band; and (iv) in any electrode channel, the scalp potential exhibited a variation larger than 150 μV within a 200 ms time window. A total of 20.18 % (SD = 10.5%) of the samples was rejected according to these criteria, without distinction for the type of stimuli (Pain-1PP: 20.76%, Pain-3PP: 19.57%; NoPain-1PP: 21.46%; NoPain-3PP: 18.94%). Moreover, a participant was rejected if each block contained at least 50% of noise. With respect to this criterion, a third participant was removed from the subsequent analyses. Next, a spherical spline interpolation process (Tikhonov regularization) was applied to the remaining data samples. The extraction of the 25 Hz energy band frequency was then performed on EEG data by applying complex Gaussian Morlet’s wavelets in order to produce time-frequency maps of the SSSR corresponding to the 25 Hz vibrotactile stimulation in a time interval. Notice that the combination of these two steps requires to reject either the whole sample or the whole electrode each time a faulty sample is found in one electrode, which leads to an increase in the number of rejected samples (Voisin et al., 2011b,c). As a final control of quality, samples with oscillatory activity over > 600 μV^2^ and any trial those reconstructed from less than 70% of the original raw data were rejected from subsequent analyses. At the end of this pre-processing, 29.8% (SD = 17.6%) of data were rejected.

Determination of the a priori region of interest (ROI) was similar to Voisin et al. (2011a,b,c) and Marcoux et al. (2013). The grand mean of the signal (i.e., blind to the actual experimental conditions) of five combinations of three surrounding electrodes over the parietal cortex were examined using paired *t*-tests during the last 200 ms pre-stimulus (i.e., during the fixation cross) versus the 200 ms time bins during the first picture presentation (i.e., before the subject could identify the condition). This approach, similar in spirit to a localisationer run in fMRI, was conducted specifically to select which group of three electrodes showed the highest gating response to the vibrotactile stimulation. As the experimental condition is not used as a criterion, the procedure has no impact on the statistical tests for the visual perspective. SSSR during the overall time course was divided in 200 ms wide time bins sampled every 100 ms that were used for the subsequent analyses to detect differences in the modulation with higher accuracy.

To assess the impact of the experimental conditions on SSSR modulation, a statistical analysis similar to that described in Decety et al. (2010) and Li and Han (2010) was used. Namely, the statistical analyses were conducted separately on two time windows for which the experimental conditions differed. In first initial gating window, no indication of pain was present, so the analysis focused on the presence of a SSSR modulation after picture onset. This variation consisted in the difference between the last 200 ms pre-stimulus (i.e., during the fixation cross), and two 200 ms long time bins immediately following the first picture presentation (i.e., 200 to 500 ms, with 100 ms overlap). The mean energy of this initial gating window was computed with paired *t* tests.

In the second time window, which refers to the specific gating, the raw SSSR was normalized to its corresponding baseline (the last 200 ms portion of the second picture, i.e., before the painful picture outcome apparition), using the following equation: (SSSR-baseline)/baseline (see Marcoux et al., 2013). Then, mean of SSSR ratios of all 200 ms time bin (total of 9 consecutive time bins) within the specific gating window (1200–2200 ms) were computed in order to systematically assess the visual perspective effect (1PP*3PP) during pain observation using paired *t*-tests. *P*-values are reported with the Bonferroni corrected alpha values. A Pearson *r* correlation analysis (two-tailed, statistical thresholds: *p* < 0.05) was performed to measure the relation between SSSR (significant time bins only) and pain ratings of painful visual stimuli in 1PP and 3PP condition. Correlation analyses were also conducted on IRI subscales and SSSR when participants were watching painful situations presented in 1PP and 3PP.

## Results

### Behavioral results

The percentage of correct responses was very high (mean 96.6%, SD = 3.3). Lower percentage of incorrect responses tends to be found in 1PP (mean = 2.8%, SD = .18) comparatively to 3PP (mean = 3.6%, SD = .21), but this difference was not significant (*t*(16) = −1.82, *p* = 0.09). A repeated measures ANOVA performed on mean pain ratings for each Perspective (1PP vs. 3PP) and Pain (Pain vs. NoPain) levels revealed no significant difference across the blocks (8) (Interaction : *F*(7, 9) = 1.77, *p* =.21), indicating that the pain ratings did not differ over time between these conditions (e.g., no significant habituation effect). Paired *t*-test performed on means of ratings of pain intensity in painful pictures showed a significant difference in Perspective condition (*t*(16) = 2.25, *p* =.02). Participants rated painful pictures in 1PP significantly higher (mean = 5.39, SD = 2.04) than those in 3PP (mean = 5.31, SD = 2.06).

### EEG results

*The initial gating* window (200 to 500 ms): The map of the cortical amplitudes in the 25 Hz band confirmed that electrodes 66, 67 and 71 (128-HydroCell Geodesic Net, Electrical Geodesic) showed the highest gating response to the vibrotactile stimulation, which corresponded to the posterior parieto-central region contralateral to the stimulation (comparable to electrodes P3-P1-PZ of the 10–20 coordinate system). The paired *t-*tests conducted on this ROI revealed a significant decrease of the SSSR amplitude values (all *ps* < .001, *α* = .03) during the display of the first picture (200 ms just after first image onset), and this suppression remained for all 200 ms time bins during the first picture presentation.

**Figure 2:**
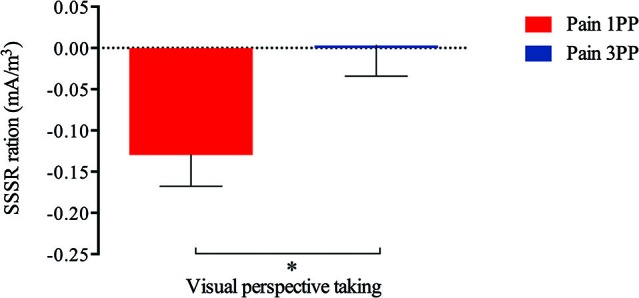
**A significant difference (*t*(16) = −2.89, *p* = .005, *α* = .006) was detected according to visual perspective after the painful outcome onset (i.e., while third picture presentation; 1900 to 2100 ms)**. More specifically, painful stimuli seen in 1PP produced a higher SSSR decrease (*M* = −.13, SD = .15) than those observed in 3PP (*M* = .003, SD = .15).

*The visual PT effect* (specific gating window, i.e., 1200 to 2200 ms): A significant effect of the Perspective condition on the SSSR was found for the 1900 to 2100 ms period (i.e., 900 and 1100 ms after the onset of the third picture) (*t*(16) = −2.89, *p* = .005, *α* = .006). The mean of SSSR ratios showed a larger decrease for painful pictures depicted in 1PP relative to those in 3PP. No significant effect of visual perspective was found in the other SSSR 200 ms time bins during the specific gating window (1200–1400 ms: *t*(16) = −.98, *p* = .17, 1300–1500 ms: *t*(16) = −1.24, *p* = .12, 1400–1600 ms: *t*(16) = −1.41, *p* = .09, 1500–1700 ms: *t*(16) = −.06, *p* = .48, 1600–1800 ms: *t*(16) = .15, *p* = .44, 1700–1900 ms: *t*(16) = −.19, *p* = .43, 1800–2000 ms: *t*(16) = −1.01, *p* = .17, 2000–2200 ms: *t*(16) = −2.07, *p* = .03, all *α* = .006), although some tendencies were found which did not survive the correction for multiple tests. Notice that, although known too severe, Bonferroni correction here reaches the same conclusion as more powerful correction such as Holm-Bonferroni method. SSSR modulation differences between conditions are presented in Figure 2.

### Correlation analyses

No significant correlations were found between SSSR initial gating and IRI subscales (EC: *r* = .47, *p* = .06, PD: *r* = −.3, *p* = .34, PT: *r* = −.33, *p* = .2, F: *r* = .03, *p* = .92). Ratios of individual SSSR decrease during observation of painful pictures in 1PP and 3PP (i.e., during the 3rd picture presentation) were not significantly correlated neither with corresponding pain ratings (1PP: *r* = −.11, *p* = .63; 3PP: *r* = −.12, *p* = .64), nor with other IRI subscales (1PP: EC: *r* = −.03, *p* = .91, PD: *r* = .09, *p* = .73, PT: *r* = −.17, *p* = .52, F: *r* = −.01, *p* = .96; 3PP: EC: *r* = −.2, *p* = .94, PD: *r* = .2, *p* = .43, PT: *r* = .21, *p* = .43, F: *r* = .15, *p* = .59).

## Discussion

The present study demonstrated, for the first time, that the visual perspective from which pain is observed could influence both the modulation of a somatosensory response and subjective pain evaluation. Using steady-state EEG, this study revealed differences in SSSR according to the visual perspective through which pain was observed in others. The results also confirmed the hypothesis that viewing pseudo-dynamic pictures in 1PP produced higher ratings of pain intensity relatively to those in 3PP. However, the absence of a significant relation between SSSR and subjective ratings of painful visual stimuli could suggest that these responses to pain observation may be underpinned by different empathic constructs.

### An overview on the somatosensory cerebral modulation

As hypothesized, the results revealed a strong general gating appearing early at the visual stimuli onset before being specific to pain observation. Given that the visual stimuli were the same for all conditions before the pain outcome onset, this general gating, found in overall somatosensory activation, is not necessarily specific to pain, but rather to observed hands in action. This result is consistent with the hypothesis that somatosensory gating reflects an attention filtering process (Cromwell et al., 2008). This “gating” effect might represent an attention filtering process that rejects incoming irrelevant somatosensory information to focus on those that are motivationally relevant (Montoya and Sitges, 2006).

### The effect of visual perspective during pain observation

Pain intensity ratings may vary according to the visual perspective from which painful pictures are presented. When pain is seen with self-proximity, as in 1PP, it is perceived as more intense than when it is watched in another’s viewpoint. Moreover, a higher tendency to make incorrect evaluation of pain intensity came out when people viewed pictures in 3PP. So, both cerebral and behavioral findings support the hypothesis that watching pain in one’s own viewpoint enhanced neurophysiological activity and pain intensity judgments. Interestingly, participants declared to have noticed different perspectives in the visual stimuli although they were not directly required to take self or other’s perspective. In other words, this point suggests that participants did not ignore the orientation of visual stimuli while judging pain intensity.

The current study also demonstrated that observing pain from a 1PP or 3PP influences the somatosensory neuronal activity. As mentioned previously, to understand another person’s visual perspective, one has to transpose the other’s spatial image onto the self perspective (Kozhevnikov and Hegarty, 2001; Kessler and Thomson, 2010). Thus, in either 1PP or 3PP visual perspectives, people have to mentally simulate an egocentric visual representation of the context seen. Similarly, a specific somatosensory modulation was found when the participants were rating pain intensity presented in painful pictures in both visual perspectives. However, a stronger SSSR decrease was found in 1PP relative to 3PP when the participants were evaluating the pain intensity seen in painful scenarios.

These results suggest that painful situations observed in a visual perspective consistent with one’s own engage to a greater extent the sensory processes of pain perception comparatively to situations seen from another’s person point of view. These findings are consistent with a previous study that has showed that changing the context from which one imagines pain (pain in self compared to pain in others) influences the level of activity in the secondary somatosensory cortex (Jackson et al., 2006b). The sensory-discriminative dimension of pain encodes the main properties of an actual painful sensation such as stimulus localization, intensity and quality discrimination (Treede et al., 1999). Thus, higher somatosensory gating effect may suggest that looking at painful situation from our point of view induces a greater encoding of the stimulus properties. In line with this result, an early review of brain imaging paper on pain perception has suggested that the pattern of activity within different regions is closer to what is found during nociception when the pain is referenced to the self as opposed to another person (Jackson et al., 2006c). This pattern of neural response may be more closely linked to the actual pain experience (Derbyshire, 2000; Jackson et al., 2006c). As mentioned previously, the general somatosensory gating is related to observation of action displaying hands before seeing the painful outcome. Therefore, an alternative hypothesis is that these results might suggest an advantage of 1PP for action understanding that consequently lead to enhanced pain perception.

One interpretation for the finding that less SSSR decrease was found in painful pictures observed in another’s person of view is that 3PP involved different cognitive processes (e.g., complex spatial transformations) to mentally rotate the stimuli to an egocentric perspective (van der Heiden et al., 2013). In accordance with this suggestion, Li and Han (2010) reported a decrease of the event-related brain potentials amplitude when changing cognitive perspective during pain observation in the late top-down controlled component but not the early automatic component. Their results indicated that pain observation initially modulates the ERP response whether the participants had to imagine that they were in a painful situation or that an unfamiliar person was in the same painful situation. Cognitive perspective processes later reduces this neural response to observed pain (Li and Han, 2010). The timing of the SSSR variation could be an interesting variable to assess precisely in future research. Altogether, these findings demonstrate that the general process of visual PT is associated with a common somatosensory neuronal response pattern. However, distinct processes are engaged when one has to evaluate pain situations observed according to the visual perspective i.e., in first- or third-person visual perspective.

Taken together, the present findings support the hypothesis that visual PT yields higher cognitive processes, and modulates the somatosensory neural activity in pain observation. However, no significant results support the relationship between pain intensity ratings and EEG data. Some studies also failed to detect significant statistical correspondence between behavioral and cerebral measures (Danziger et al., 2009; Voisin et al., 2011a). Thus, it is reasonable to suggest that seeing and evaluating pain might engage distinct constructs. Further, this leads to the idea that other behavioral measures, such as response latency, could probably be more related to time-frequency neurophysiological data and should be considered in future work.

Some limitations need to be addressed in this study. First, our sample size was relatively small, reducing the quantity of EEG and behavioral data. To overcome this inconvenience, we used a specific EEG pre-processing that keeps an optimal amount of EEG data for analyses. Second, we did not include neuropsychological tests, so a comparison could not be made for possible interaction between visual perspective abilities and specific cognitive functions (e.g., inhibition). Third, the type of strategy that participants used to evaluate pain intensity was not controlled in the experiment. Someone who evaluates pain intensity based of the visual perspective could give different ratings between 1PP and 3PP, while another person could refer to his personal experience, regardless of the orientation of stimuli. These current limitations should be considered as possible avenues for future research on visual PT.

## Conclusion

The neuronal and behavioral mechanisms of visual PT were examined in a pain observation paradigm, a widely recognized methodology for the study of different components of empathy (Decety et al., 2006; Fitzgibbon et al., 2010; Lamm et al., 2011). The present results demonstrated that seeing pain from self- or other- visual perspectives produce partly similar neuronal responses, which enable a person to share and to understand another’s person pain experience even if it is different from his or her point of view. This study further illustrated that the characteristics of the somatosensory cerebral modulation could differ between self and other’s visual perspective. The current study lays the basis for further studies on pain communication where the consideration of different points of view can be influenced by the visual perspective from which a situation is perceived. We also emphasize the relevance for further investigations using a similar experimental paradigm with psychiatric populations, such as schizophrenia, in which general PT deficits are observed (Langdon et al., 2006; Montag et al., 2007; Derntl et al., 2009).

## Conflict of interest statement

The authors declare that the research was conducted in the absence of any commercial or financial relationships that could be construed as a potential conflict of interest.
